# Oral Mucosa Status in Patients with End-Stage Chronic Kidney Disease Undergoing Hemodialysis

**DOI:** 10.3390/ijerph20010835

**Published:** 2023-01-02

**Authors:** Elżbieta Dembowska, Aleksandra Jaroń, Ewa Gabrysz-Trybek, Joanna Bladowska, Grzegorz Trybek

**Affiliations:** 1 Specialized Center of Dentistry-Elzbieta Dembowska, al. Bohaterow Warszawy 11b/5, 70-370 Szczecin, Poland; 2 4th Military Clinical Hospital in Wroclaw, ul. Rudolfa Weigla 5, 50-981 Wroclaw, Poland; 3 Department of Oral Surgery, Pomeranian Medical University in Szczecin, 70-111 Szczecin, Poland; 4 Individual Specialist Medical Practice Ewa Gabrysz-Trybek, 70-111 Szczecin, Poland; 5 Department of General and Interventional Radiology and Neuroradiology, Wroclaw Medical University, M. Curie-Skłodowskiej 68, 50-369 Wrocław, Poland

**Keywords:** oral mucosa, oral pathologies, end-stage renal disease, chronic kidney disease, hemodialysis

## Abstract

There are reports in the literature of interrelationships between chronic kidney disease and periodontitis pathophysiology; similar risk factors play a role in these conditions. Due to chronic kidney disease (CKD), patients on hemodialysis (HD) are more susceptible to developing pathological processes in the gingiva, periodontium, and oral mucosa. This study aimed to evaluate the condition of the oral cavity, with particular attention to lesions of the oral mucosa of patients with end-stage renal disease in Poland, West Pomeranian Voivodship. A case-control study assessed oral health in 200 Polish subjects, including 100 dialysis-station patients who constituted the study group (HD) and 100 healthy patients who formed the control group (K). The physical examination consisted of a general medical and dental history. Evaluation of the oral mucosa included detailed noting of the type of lesions, nature of complaints, and their location. The results showed a higher prevalence of oral lesions highlighting oral mucosal pathology in patients with HD than in group K. Most common symptoms reported by patients with CKD (HD) were xerostomia, taste disorders, and burning mouth. These findings highlight the need to implement comprehensive multispecialty care in patients with chronic systemic diseases.

## 1. Introduction

Oral diseases affect more than 2% of the population [1]. The oral mucosa of patients with end-stage renal disease (CDK) becomes susceptible to the occurrence of pathologic lesions and metabolic abnormalities due to water-electrolyte anomalies, in addition to continuous irritation by toxic metabolites associated with the general disease that are present in their saliva, as well as a predisposition to xerostomia (Table 1) [2]. Chronic kidney disease (CDK) patients are often prone to periodontitis and inflammation in the oral mucosa [3]. 

Adequate bone metabolism and oral cavity physiology are influenced by calcium–phosphate balance [4]. 

However, there are reports in the literature that there is no effect of portal hyperparathyroidism in hemodialysis patients on the periodontal status and the bone level of the alveolar processes of the maxilla and mandible [5].

In chronic kidney failure, oral mucosal abnormalities may take nonspecific and disease-specific forms [4]. Among nonspecific symptoms of chronic kidney disease (CKD), white and red oral mucosa lesions in leukoplakia and erythroplakia have been described. Lichenoid lesions have also been reported due to disease and pharmacotherapy [4]. There is a known correlation between using diuretics and beta-blockers and developing lichen planus or lichenoid lesions in the oral mucosa [5]. Ulcerations, fibromas, and papillomas are also frequently observed in patients with CKD and papillomas. Black or hairy tongue and geographic lesions have been observed in the tongue area. Immune-system impairment accompanying chronic kidney failure may contribute to developing viral infections and hairy leukoplakia [6]. A specific mucosal manifestation of renal dysfunction may be the so-called uremic stomatitis reported in this group of patients. Cases of uremic stomatitis mimicking the appearance of hairy leukoplakia on the dorsal and lateral surfaces of the tongue have been reported in the literature [6].

In the literature, similar studies are observed in populations other than the one studied by the authors. The authors review the population of Poland, West Pomeranian Voivodship. We have not yet found a study on this issue in the Polish population. The literature contains, among others, studies on populations of Iran [7], Iraq [8], Italy [9], and India [10].

This study aimed to evaluate the condition of the oral cavity, with particular attention to lesions of the oral mucosa of patients with end-stage renal disease in Poland, West Pomeranian Voivodship.

## 2. Materials and Methods

In our case-control study, oral mucosa status was assessed in 200 subjects, including 100 dialysis-station patients, who constituted the study group, and 100 healthy patients, who formed the control group. The study was conducted after obtaining the consent of the Bioethics Committee of the Medical University (No. K0012/45/11). The study was preceded by obtaining informed consent of the subjects to participate and to publish it, and they were informed in detail about its purpose and course.

Study group (HD): *n* = 100, hemodialyzed patients with end-stage renal disease.

Control group: *n* = 100, healthy subjects.

The control group was selected to be adequate concerning age and sex distribution to the control group.

The control group consisted of 100 completely healthy patients who attended the Periodontics Clinic for hygiene visits. They did not suffer from any diseases and did not take any medications.

Statistical analysis of mean age in both groups showed no statistically significant differences (*p* = 0.77).

The following inclusion and exclusion criteria were adopted in the study.

Study group (HD) inclusion criteria: dialysis for at least three months, informed consent to participate in the study, and an end-stage renal disease requiring hemodialysis. Patient exclusion criteria: cancer, taking cytotoxic or immunosuppressive drugs, antibiotic therapy at the time of the study or within the last three months.

### 2.1. Physical Examination

The subjective examination consisted of a general medical and dental history.

Two periodontology and mucosal disease specialists evaluated oral cavity status; in case of discrepancies, a third physician evaluated the lesions. In the initial study, both physicians were calibrated by examining ten patients. In 100% of cases, the physicians made the same diagnoses.

Information was collected from patients on general health status, comorbidities, and duration of dialysis therapy. Data on the patient’s weight and height were also obtained based on the interviews to assess body mass index (BMI) [11]. This index, which is an anthropometric parameter used to evaluate a patient’s nutritional status [12], is expressed in kg/m², calculated based on the following formula:

(1) $$\mathrm{BMI}\;\left[\frac{\mathrm{kg}}{m^{2}}\right]=\frac{\mathrm{body}\;\mathrm{mass}\;\left[\mathrm{kg}\right]}{\mathrm{height}\;{\left[m\right]}^{2}}.$$

It is advisable for the accuracy of BMI calculations in patients undergoing hemodialysis to use the numerical value of dry body weight [12,13]. According to many authors, dry body weight is an adequate reference point for BMI calculations in dialysis patients. The possibility of fluid body retention and the resulting increase in body weight between dialysis treatments may distort the adequacy of the calculated index [13,14]. One of the definitions used in nephrology presents dry body weight as the weight gained after a given hemodialysis session, accompanied by an average blood-pressure level maintained until the next hemodialysis session despite sodium retention [14,15]. In the study described herein, for comparative purposes, we obtained anamnesis information about the dry body weight of a dialyzed patient (measured after hemodialysis). Two interpretative divisions of BMI were used in this study: one analogous to the detailed division proposed by WHO [12,15]; and a simplified one, taking into account the presence or absence of overweight on non-overweight patients presenting a BMI value < 25 kg/m² and overweight or obese patients corresponding to a BMI value ≥ 25 kg/m².

### 2.2. The Condition of the Oral Mucosa in the Study Groups

Assessment of oral mucosal conditions included detailed noting of the type of lesions, nature of complaints, and their location (Table 2). A direct oral swab was taken and underwent a mycological examination to confirm mycosis. In the case of lesions, specimen sections were taken for histopathological examination to confirm or negate the clinical findings. The prevalence of oral mucosal conditions and lesions in the study groups and the number of retained teeth were assessed. In the case of recurrent lesions, patients came for follow-up examinations.

### 2.3. Statistical Analysis

The Kolmogorov–Smirnov test was used to determine the normality of the distribution of the variables. Variants were characterized using means, standard deviations, and outliers. Student’s t-test and Mann–Whitney tests were used to examine differences between the study groups (HD, K). Using frequency and number of occurrences, discontinuous variables were described, and relationships were characterized using Pearson’s χ^2^ (chi-square) test. We also used the evaluation of relative risk–odds ratio (OR) and 95% confidence interval (CI) and probability to assess the risk of conditions in the context of different causes and the logistic regression model. Statistically significant differences presented a confidence level of *p* < 0.05. Statistical analysis was performed using the STATA 11 program license number 30110532736.

## 3. Results

### 3.1. Baseline Characteristics

Table 3 shows the details of the age of the subjects.

The study also analysed BMI in both groups (HD. K). BMI below 25 kg/m^2^ was found in 44% (*n* = 44) of HD and 42% (*n* = 42) of K patients. BMI equal to or higher than 25 kg/m^2^ was present in 56% (*n* = 56) of HD and 58% of the K group. There were no statistically significant differences between the representatives of both groups (*p* > 0.05).

The evaluation of mean BMI values for both genders showed statistically significant differences in the hemodialysis (HD) group (*p* = 0.004), where the mean BMI value for men (*n* = 57) was 26.22 (±3.13) and for women (*n* = 43) was 24.22 (±3.65). There were no statistically significant differences in the control group (K) (*p* = 0.702), in which the mean BMI values for men (*n* = 58) and women (*n* = 42) were 25.60 (±2.72) and 25.32 (±2.72), respectively.

Table 4 shows the diseases that the subjects suffered from in addition to CKD.

### 3.2. Oral Health

A statistically significantly higher frequency of oral mucosal lesions was found in the study group (HD) compared to the control group (K) (*p* < 0.0010). Oral mucosal lesions were found in 52% (*n* = 52) of the HD group and only 2% (*n* = 2) of the K group.

Analysis of the prevalence of oral mucosal conditions (Table 5) in the HD group revealed that xerostomia was the most common. observed in 22% of the subjects in the HD group and in 0% of the subjects in the K group (*p* < 0.001). Next in terms of frequency of occurrence was taste disorder. observed in 14% of subjects in the HD group and 1% of subjects from group K (*p* = 0.0004). The following frequency of occurrence was burning mouth. which occurred in 11% of the subjects in the HD group and none of the subjects in group K (*p* < 0.001). Mycosis detected by mycological examination was noted in 5% (*n* = 5) of hemodialysis patients (*p* = 0.024). Herpes labialis was found in 4% (*n* = 4) of hemodialysis patients, and in 2% of cases. it was found in the corners of the mouth. A similar frequency of white lesions was observed in the HD group—2% (*n* = 2). A nodular lesion of undetermined character was observed in 1% (*n* = 1) of the HD group. Black hairy tongue was present in 1% of the HD group. In 1% (*n* = 1) that was lesions on the trauma background. White lesions were observed in 1% (*n* = 1) in the K group. Statistically significant differences were found in the incidence of xerostomia, taste disorders, burning mouth, tinea capitis, and herpes labialis (*p* < 0.05). There were no statistically significant differences in the frequency of other conditions (*p* > 0.05).

**Figure 1:**
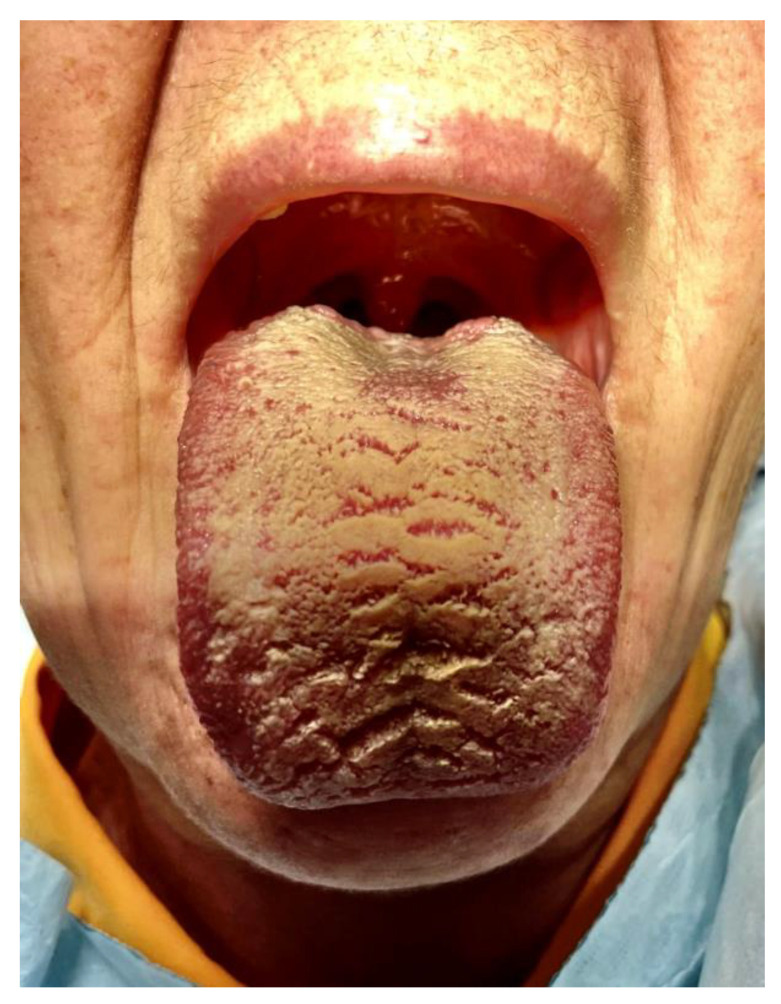
Hairy tongue.

**Figure 2:**
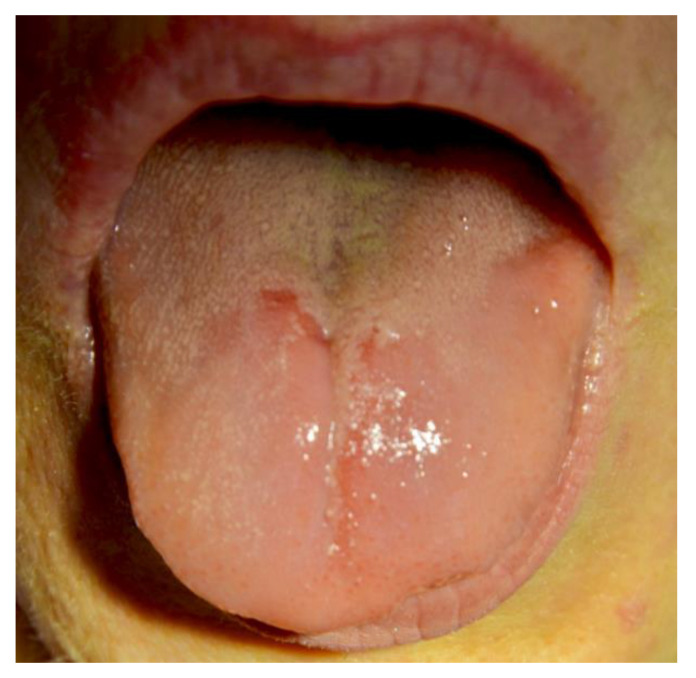
Oral atrophic mucositis.

**Figure 3:**
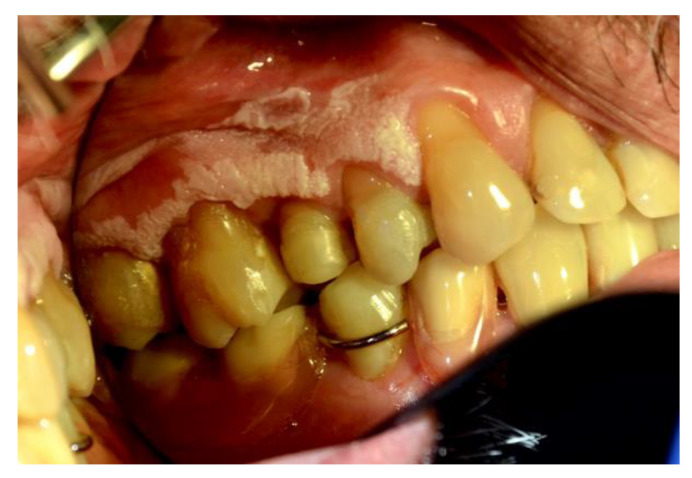
Hyperkeratosis.

**Figure 4:**
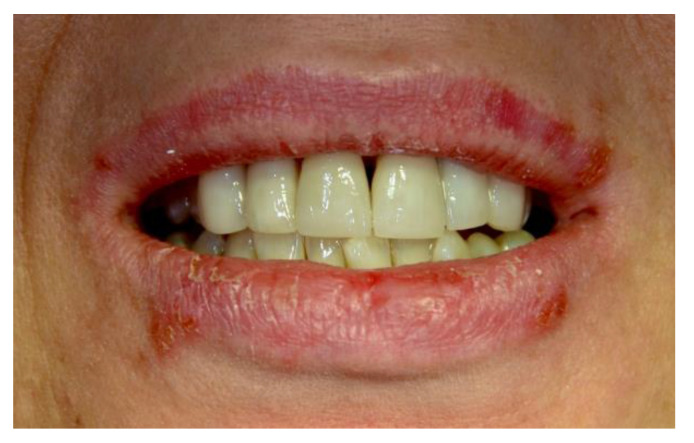
Recurrent herpes labialis.

**Figure 5:**
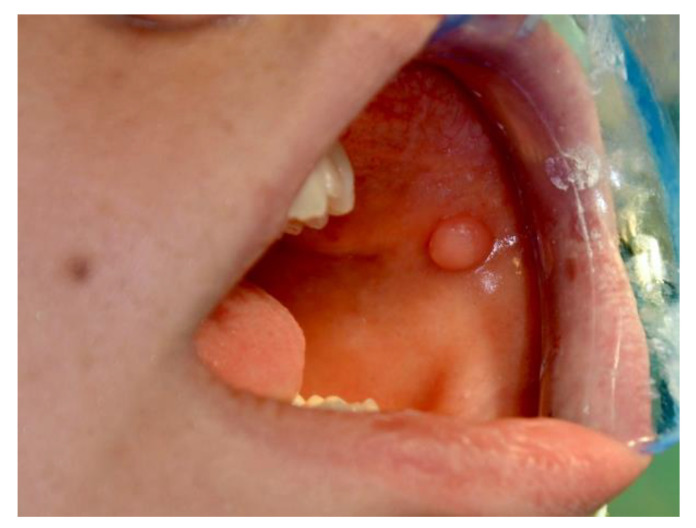
Fibroma.

**Figure 6:**
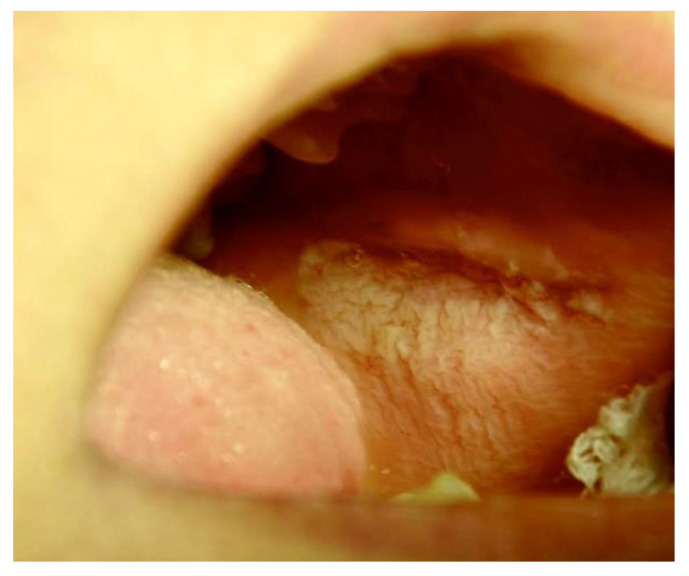
White lesion.

Figure 1, Figure 2, Figure 3, Figure 4, Figure 5 and Figure 6 show examples of conditions of the oral mucosa in the study groups.

No statistically significant correlation was found between the occurrence of oral mucosal lesions and diseases and: age; BMI; and duration of dialysis in the study group (HD) (Table 6).

As shown in Table 7, a statistically significant relationship was noted between the occurrence of recurrent herpes and BMI in the hemodialysis (HD) group (*p* = 0.008). There was also a statistically significant association between the occurrence of recurrent herpes with higher mean gingival index (GI) values by Löe and Sillness in the hemodialysis (HD) group (*p* = 0.0035).

A risk assessment of oral mucosal disorders and conditions was also conducted among hemodialysis (HD) patients (compared to the control group (K). There was a 50-fold lower chance of keeping the oral mucosa healthy (OR 0.02; 95% CI 0.00–0.08). The likelihood of oral mucosal candidiasis was more than five times higher in the study group (HD) compared to the control group (OR 5.26; 95% CI 1.34–251.33). There was also a 28 times higher risk of xerostomia in the HD group compared to the K group (OR 28.21; 95% CI 7.26–1175.17). A 16-fold higher risk of taste disturbance in the HD group compared with the K group was also described (OR 16.12; 95% CI 2.08–125.09). The risk of recurrent herpes zoster in hemodialysis patients was defined as more than four times higher compared to the control group (OR 4.21; 95% CI 1.07–209.36).

The number of teeth retained in the oral cavity of patients in both groups was analysed. The number of ≥20 teeth preserved in the oral cavity was found in as many as 75% (*n* = 75) of patients in the control group (K) and only 47% (*n* = 47) of dialysis patients (HD). In the range of 7–19 retained teeth in the oral cavity. 23% (*n* = 23) of patients of the control group (K) and 44% (*n* = 44) of the hemodialysis patients (HD). In contrast. only 1–6 teeth retained in the oral cavity were found in 2% (*n* = 2) of the control group and 9% (*n* = 9) of the study group (HD). Statistically significant differences were found in the number of teeth retained in the oral cavity between the two study groups (*p* < 0.001). The results discussed above are shown in Table 8.

There was a significant statistical difference between the evaluated groups (*p* < 0.001) for the mean number of retained teeth. In the HD group. the mean number of preserved teeth in the oral cavity is 18.08 (±7.50), while in the K group, it is 23.25 (±6.25). The table shows the statistical parameters of the values of the number of teeth present in the oral cavity in the studied groups (HD, K), also noting the division by gender. No statistically significant differences were found for the number of teeth in the context of the sex of the subjects in both groups (*p* > 0.3).

## 4. Discussion

Chronic kidney disease (CKD), especially all the pathophysiological processes occurring in its end-stage, leads to various oral changes. The oral anatomy and morphology, which are extremely complex, make it possible for these changes to affect the periodontium, teeth, and oral mucosa. Malnutrition is widespread in CKD patients due to impaired taste and smell, leading to weight loss [2,16]. In addition. these patients have dry mouths, making food intake difficult. This is due to impaired salivary gland function in CKD patients [2,16,17,18].

The taste sensitivity threshold may be positively influenced by low zinc levels present in the patient’s organism in saliva, serum, and leukocytes [19].

The occurrence of taste disorders is also influenced by high urea concentration, which is observed in patients with CKD [16]. Another unpleasant symptom is halitosis, i.e., bad breath [20]. Hemodialysis patients are more susceptible to oral pathogens. Many pathogens are present in these patients, including Prevotella intermedia, Porphyromonas gingivalis, Aggregatibacter actinomycetemcommitans, and Candida albicans [21].

In peritoneal dialysis patients. periodontitis is much more common than in healthy patients and is also associated with poorer patient nutrition (malnutrition) and higher inflammatory markers in the blood [22].

Parallel to the pathophysiological conditions that promote the development of periodontal tissue disorders. in a group of patients with CKD. abnormal health-promoting behaviors and unhealthy behaviors result in a higher incidence of periodontitis. Among these habits, a lack of regularity in performing oral hygiene procedures has been described in many studies [23,24,25].

In the present study. oral mucosal lesions were found to be significantly more common in hemodialysis patients (52%) compared to the control group (2%). Xerostomia was observed in 22% of hemodialysis patients and in no one in the control group. Taste disorder was represented by 14% of hemodialysis patients and 1% of the control group. Burning mouth was reported in 11% of the patients. Mycosis was reported in 5% of hemodialysis patients.

The mucosa of hemodialysis patients with end-stage renal failure shows a marked tendency for pathological changes. There is a higher incidence of taste disorders, xerostomia, and other conditions in this group of patients [26].

Thorman et al. [27] also noted that mucosal changes such as lichen planus, atrophic oral mucositis, ulcers, and various infections were more frequently observed in the group with end-stage MS. A study by Malekmakan et al. [28] observed a high prevalence of xerostomia (48.6%) and taste disorders (49.3%) in hemodialysis patients.

Similarly, Chuang et al. [29] found a higher prevalence of xerostomia, taste disorders, and burning mouth in hemodialysis patients with diagnosed diabetes compared to HD patients without diabetes. The frequent occurrence of oral fungal infections may be related to the higher prevalence of Candida albicans in saliva in hemodialysis patients than in the control group [21].

Baranowicz-Gąszczyk et al. [30] reported a higher number of lost teeth in hemodialysis patients compared to the control group (*p* < 0.001). The range of up to 5 lost teeth included 21.4% of the general population studied and 3.4% of hemodialysis patients. On the other hand, a number between 16 and 31 lost teeth was recorded in 10.7% of the studied general population and as many as 41.4% of hemodialysis patients (*p* < 0.001). The authors also observed a correlation between the nutritional status of hemodialysis patients and dental status. Compared to the general population. hemodialysis patients are characterized by a poorer prosthetic supply [21]. Marinho et al. [25] observed higher plaque accumulation, more teeth with clinical attachment loss, and a higher number of lost teeth in patients with chronic kidney disease than in controls.

There is a two-way relationship between the occurrence of periodontitis and general diseases. Periodontal diseases modify the course of diseases such as hypertension and diabetes. Furthermore, Desvarieux et al. [31] indicated that periodontitis might predispose patients to hypertension. It has also been shown that the prevalence of periodontitis is significantly higher among middle-aged individuals with diabetes than among similarly aged individuals without diabetes [32]. For such patients. prophylactic measures. frequent clinical check-ups to rule out oral lesions, prevention, and patient education about the possibility of oral lesions, are extremely important.

It should be taken into account that chronic diseases affect the wuality of life (QoL) of patients, so special attention must be paid to prevention to prevent patients’ QoL from declining [33,34].

This study led to some thoughts and the creation of clear guidelines for a holistic approach to patients. First and foremost, patients with kidney disease, especially those on hemodialysis, should be pre-sanitized, followed by regular oral examinations—every 1–3 months, to identify and implement treatment of oral lesions as soon as possible.

The above study has some limitations. Due to the difficulty of collecting the study group, sample-size calculations were not done, but convenience sampling was used. All available individuals within the specified time frame who met the inclusion criteria were included in the study. To this number, a control group was selected that matched the study group in terms of age and gender. Unfortunately, the study did not determine the cause of CKD. Due to difficult access to the study subjects and their comfort related to hemodialysis, additional tests were waived by the patients, such as oral swabs, determination of saliva pH, saliva secretion rate, and buffer capacity. In future studies, we plan to perform these additional tests. In addition, we plan to establish a preventive program for hemodialysis patients related to oral health maintenance and check-ups.

## 5. Conclusions

The results showed a statistically higher prevalence of oral lesions, emphasizing oral mucosal pathology, such as taste disorders, xerostomia, and other conditions, among HD patients than in the K group. These results highlight that it is necessary to implement comprehensive multispecialty care in patients with chronic systemic diseases.

## Figures and Tables

**Table 1:** Dental abnormalities resulting from chronic kidney disease, according to Kho et al. [2].

Nature of Disorder	Prevalence
Uremic odor	34.1%
Xerostomia	32.9%
Taste disorders	31.7%
Pain in the tongue and oral mucosa	12.2%
Petechiae	12.2%
Tongue coating	12.2%
Enamel hypoplasia	3.7%
Oral mucosal ulcers	1.2%

**Table 2:** Parameters considered in the assessment of oral mucosa.

Parameters to Be Considered in the Evaluation of Oral Mucosity
Oral Mucosal Burning
Xerostomia
White lesions
Recurrent aphthae
Traumatic ulcers
Inflammation of the corners of the mouth
Mycosis (confirmed by mycological examination)
Hairy tongue
Recurrent herpes labialis
Oral atrophic mucositis
Changes due to chronic mechanical irritation
Taste disorders

**Table 3:** Summary of mean and extreme age values and BMI in both study groups.

		Study Group (HD)		Control Group (K)		*p*
AGE	Mean age	55.18 (±16.43)		52.58 (±15.46)		0.2207
	Minimum age	19.00		18.00		
	Maximum age	85.00		83.00		
	Q25	43.00		40.50		
	Q75	67.00		64.50		
	Mean age according to sex	Female	Male	Female	Male	
		54.88	55.40	52.67	52.50	
BMI	<25.00	44		42		86
	≥25.00	56		58		114
	χ^2^ Pearson	0.08		df = 1		*p* = 0.776

**Table 4:** General diseases in the study group.

Disease	Prevalence	Study Group (HD)
Hypertension	Yes	72
	No	28
Diabetes	Yes	29
	No	71
Coronary artery disease	Yes	14
	No	86
Osteoporosis	Yes	11
	No	89
Glomerulonephritis (CLN)	Yes	9
	No	91
Rheumatoid arthritis (RA)	Yes	9
	No	91
Endocrine disorders	Yes	5
	No	95
Hyperparathyroidism	Yes	4
	No	96
Hepatitis B and C	Yes	4
	No	96
Psychiatric disorders	Yes	6
	No	94

**Table 5:** Characteristics of changes and disorders of the oral mucosa in the studied groups (HD, K).

Presence of Mucosal Lesions	Study Group (HD)	Control Group (K)	Total	
Yes	52	2	54	
No	48	98	146	
χ^2^ Pearson	63.42	100	*p* < 0.001	df = 1
Characteristics of disorders and changes in the oral mucosa				χ^2^ Pearson
Xerostomia	22	0	22	*p* < 0.001
Taste disorders	14	1	15	*p* < 0.05
Burning mouth	11	0	11	*p* < 0.001
Mycosis	5	0	5	*p* < 0.05
Herpes labialis	4	0	4	*p* < 0.05
Inflammation of the corners of the mouth	2	0	2	*p* = 0.155
White lesions	2	1	3	*p* = 0.561
Aphthous lesions	1	0	1	*p* = 0.316
Traumatic lesions	1	0	1	*p* = 0.316
Black hairy tongue	1	0	1	*p* = 0.316
Tumors	1	0	1	*p* = 0.316
Occurrence of mucosal disorders	OR	95%	CI	*p*
Xerostomia	28.21	7.26	1175.17	0.00
Taste disorders	16.12	2.08	125.09	<0.05
Mycosis	5.26	1.34	251.33	<0.05
Recurrent herpes labialis	4.21	1.07	209.36	<0.05
No oral mucosal disorders	0.02	0.00	0.08	<0.001

**Table 6:** Prevalence of oral mucosal lesions vs. age. dialysis duration and BMI values in the study group (HD).

Oral Mucosal Lesions	Age								
	N	Mean	±SD	Q25	Me	Q75	*p*	r^2^	r
Yes	52	56.48	15.03	45.50	58.00	66.00	0.413	0.01	0.08
No	48	53.77	17.89	40.00	55.00	68.00			
	Dialyzis time								
Yes	52	51.19	55.51	12.00	24.00	66.00	0.099	0.03	0.17
No	48	34.94	40.22	6.00	21.00	48.00			
	BMI								
Yes	52	25.76	3.84	23.13	25.44	27.43	0.427	0.01	0.08
No	48	25.19	3.24	22.09	25.72	27.73			

**Table 7:** Incidence of recurrent herpes and BMI and GI in the study group (HD).

Presence of Recurrent Herpes	BMI								
	N	Mean	±SD	Q25	Me	Q75	*p*	r^2^	r
No	95	25.30	3.18	22.79	25.71	27.55	<0.05	0.07	0.27
Yes	4	30.09	8.38	23.53	29.83	36.65			
	Gingival Index by Löe i Sillness								
No	95	1.20	0.53	0.71	1.21	1.50	<0.05	0.08	0.29
Yes	4	2.02	0.71	1.56	1.90	2.49			

**Table 8:** Comparison of the number of teeth retained in the oral cavity in both study groups (HD. K).

Number of Teeth	Study Group (HD)		Control Group (K)		Total
≥20 teeth	47	47.00%	75	75.00%	122
7–19 teeth	44	44.00%	67	23.00%	67
<6 teeth	9	9.00%	11	2.00%	11
Total	100		200		200
χ^2^ Pearson	df = 2		<0.05		<0.05

## Data Availability

Data are available on request.
